# Pediatric central nervous system infections in the Amazon: clinical and laboratory profiles

**DOI:** 10.3389/fpubh.2023.1329091

**Published:** 2023-12-21

**Authors:** Eveny Perlize Melo Marinho, Ewerton da Silva Ferreira, Caio Cesar Leiva Bastos Barrionuevo, Sabrina Araújo Melo, Jady Shayenne Mota Cordeiro, Sergio Damasceno Pinto, Rossicleia Lins Monte, Valderjane Aprígio da Silva, Yasmin Ferreira Martins, Monique Freire Reis, Samantha dos Santos Tufic-Garutti, Vanderson de Souza Sampaio, Daniel Barros de Castro, Pablo Vinicius Silveira Feitoza, Lucia Alves da Rocha, Luiz Carlos de Lima Ferreira, Michele de Souza Bastos

**Affiliations:** ^1^Universidade Federal do Amazonas, Manaus, Brazil; ^2^Universidade do Estado do Amazonas, Manaus, Brazil; ^3^Fundação de Medicina Tropical Doutor Heitor Vieira Dourado, Manaus, Brazil; ^4^Departamento de Patologia e Medicina Legal, Universidade Federal do Amazonas, Manaus, Brazil; ^5^Departamento de Ensino e Pesquisa, Fundação Centro de Controle de Oncologia do Amazonas, Manaus, Brazil; ^6^Fundação de Vigilância em Saúde Dra. Rosemary Casta Pinto, Manaus, Brazil; ^7^Hospital e Pronto Socorro da Criança Zona Oeste, Manaus, Brazil

**Keywords:** cerebrospinal fluid, neurological infections, viral infection, adolescents, bacterial meningitis, Brazil

## Abstract

**Background:**

Central nervous system (CNS) infections are important causes of mortality and morbidity in children, and they are related to severe problems such as hearing loss, neurological sequelae, and death. The objective was to describe clinical and laboratory exam profiles of children who were diagnosed with CNS infections.

**Methods:**

We conducted a cross-sectional study based on medical records, which included pediatric patients aged from 3 months to 15 years, with a clinical suspicion of CNS infection between January 2014 to December 2019. The pathogens were confirmed in cerebrospinal fluid (CSF) samples using Gram staining, cell culture, molecular diagnostics (PCR and qPCR), and serology.

**Results:**

Out of the 689 enrolled patients, 108 (15.6%) had laboratory-confirmed infections in CSF. The most common bacterial pathogens isolated from the culture were *Neisseria meningitidis* serogroup C in 19, *Streptococcus pneumoniae* in 11, and *Haemophilus influenzae* in seven samples. The viruses identified were *Enterovirus*, *Cytomegalovirus*, Var*icella-zoster virus*, *Epstein–Barr virus*, and arbovirus. No patient was found to be positive for *Herpes simplex virus 1 and 2*. Patients with viral infections showed altered levels of consciousness (*p* = 0.001) when compared to bacterial infections.

**Conclusion:**

This study shows the presence of important vaccine-preventable pathogens, and different families of viruses causing CNS infections in the pediatric patients of Manaus.

## Introduction

Central nervous system (CNS) infections in children are considered a public health problem that causes high lethality rates, especially in developing countries (1). These infections may encompass some clinical conditions, such as meningitis, encephalitis, and meningoencephalitis, that require urgent attention and extensive investigative efforts. If CNS infection is not properly treated, it can have serious consequences in the pediatric population, leading to hearing loss, neurological sequelae, and death (2).

The epidemiology of CNS infections in children in developed countries is well documented, but in developing countries, such as Brazil, epidemiological and laboratory surveillance of these infections remains limited, leading to underestimation and underreporting of cases. Therefore, the incidence of CNS infections in developing countries is higher, with rates of 726 cases per 100,000 inhabitants, while in developed countries it is 11 per 100,000 inhabitants (3).

The main infection affecting the pediatric population is bacterial meningitis (BM) (4). The clinical and epidemiological characteristics of the infection are directly related to an interaction of socioeconomic, environmental, and infectious factors. Even though the introduction of conjugate vaccines has reduced the incidence and infant mortality rates due to BM, it remains lethal, especially in areas where vaccination coverage is limited (5). The main bacterial agents that can cause meningitis in children under 5 years of age are *Haemophilus influenzae b*, *Streptococcus pneumoniae*, and *Neisseria meningitidis* (6). In addition, Gram-negative bacteria of the Enterobacteriaceae family pose a risk to neonates (7).

CNS viral infections, including meningitis and encephalitis, contribute to the burden of pediatric neurological diseases (8). However, the burden of viral meningitis (VM) remains uncertain due to gaps in surveillance systems, especially in developing countries (4). Common viral pathogens associated with these conditions include viruses from the Picornaviridae, Herpesviridae, and Flaviviridae families (9).

Between 2014 and 2022, Brazil reported 127,215 cases of CNS infections in children with different etiologies. Among these, VM was the most prevalent with 58,872 of the cases. Although the numbers are alarming, epidemiological data on CNS infections in children in the Amazon region are scarce (10).

In this context, it is essential to understand the profile of neurological infections in the pediatric population. Therefore, active epidemiological and laboratory surveillance can contribute to the knowledge of circulating agents. Thus, this study aimed to describe the clinical and laboratory profiles of children from Manaus, Amazonas State, Brazil, who were diagnosed with CNS infections.

## Methods

### Study population

We conducted a cross-sectional study based on medical records. We studied pediatrics patients aged from 3 months to 15 years, admitted in 11 hospitals (S1 Appendix in Supplementary material) in Manaus from January 2014 to December 2019, with a clinical suspicion of CNS infection, who had presented with an acute neurological condition associated with a presence of one or more signs or symptoms, such as neck stiffness, fever, rash, altered level of consciousness, muscle weakness. Lumbar puncture to get cerebrospinal fluid (CSF) sample was performed on all the patients on the admission day by the clinicians, and the CSF was forwarded for analysis at the Bacteriology laboratory at Fundação de Medicina Tropical Dr. Heitor Vieira Dourado (FMT-HVD). We identified patients using the laboratory database, we retrospectively identified 108 patients who had pathogens detected in CSF regardless of indication. We excluded patients who had undergone surgery, suffered any kind of trauma, or had CSF collected from an external ventricular shunt (EVS) or ventricle-peritoneal shunt (VPS).

### Data collection and processing

Demographic, past medical history, admission history, examination, investigations, diagnosis, and management were obtained from electronic medical records. Laboratory tests were collected at the laboratory database. Children over 1 year with symptoms like fever, headache, vomiting, neck stiffness, seizures, or rash were considered as CNS infection cases. In children under 1 year, we also considered irritability, persistent crying, and the existence of fontanel bulging (11).

### Case definition

Confirmed CNS infection were diagnosed based on the detection of bacteria pathogens in the CSF culture, latex agglutination test, as well as the presence of bacteria following Gram staining. Confirmed cases of viral infection were characterized by the etiological agent’s confirmation through polymerase chain reaction (PCR) and the presence of dengue and/or Zika virus-specific IgM antibodies in the CSF.

### Laboratory analysis

All cases were investigated through CSF analysis according to established laboratory protocols. Biochemical, cytological, and microbiological analyses of CSF were performed according to laboratory standards. This included the evaluation of CSF glucose, protein, and lactate levels, global and differential cell count, and staining with Gram, Ziehl-Neelsen and Nankin ink. Additionally, microbiological culture were performed.

### Molecular diagnosis

For the viral investigation, 200 uL of CSF was extracted using the ReliaPrep™ (Promega) kit, following the manufacturer’s instructions. PCR was employed to detect the following viruses: herpes simplex virus 1 and 2 (HSV-1 and 2), varicella zoster virus (VZV), Epstein–Barr virus (EBV), cytomegalovirus (CMV) and parvovirus B-19 (PVB-19). Reverse transcriptase PCR (RT-PCR) was used to investigate the enterovirus, dengue virus (DENV 1–4) and Zika virus, according to the protocols described by Brazil Ministry of Health (12), Lanciotti et al. (13, 14), Markoulatos et al. (15), and Oberste et al. (16). Each reaction contained CSF samples, a positive control, a negative control (water), and an internal control consisting of β-actin and RNAse P (RP) amplification to validate the presence of nucleic acid.

### Serological diagnosis

The CSF samples (diluted 1:5) were analyzed for presence of IgM antibodies to dengue virus (17), and IgM antibodies to Zika virus were measured using a recommended capture via enzyme-linked immunosorbent assays (ELISA) based on the Centers for Disease Control and Prevention (CDC) (CDC Fort Collins, CO, United States) (18).

### Data analysis

We analyzed data statistically in BioEstat v.5.0. Categorical variables were presented as absolute and relative frequencies, while continuous variables were expressed as means and medians, considering standard deviation (SD) and range, respectively. To compare bacterial and viral groups, was used the chi-square test and odds-ratio comparisons, considering a value of p of 0.05 as statistically significant.

Software QGIS® 3.16.9-Hannover version for Windows^®^ 10, was used to construct density maps to demonstrate the spatial distribution of cases with etiological agents detected in the CSF.

### Ethics statement

This study was approved by the Ethics Review Board of the FMT-HVD under (CAAE: 12775119.5.0000.0005), following the guidelines and standards established in Resolution 466/12, of the National Health Council of the Brazilian Ministry of Health. A waiver of informed consent was granted due to the retrospective nature of the study, and patient-identifying data was anonymized.

## Results

**Figure 1:**
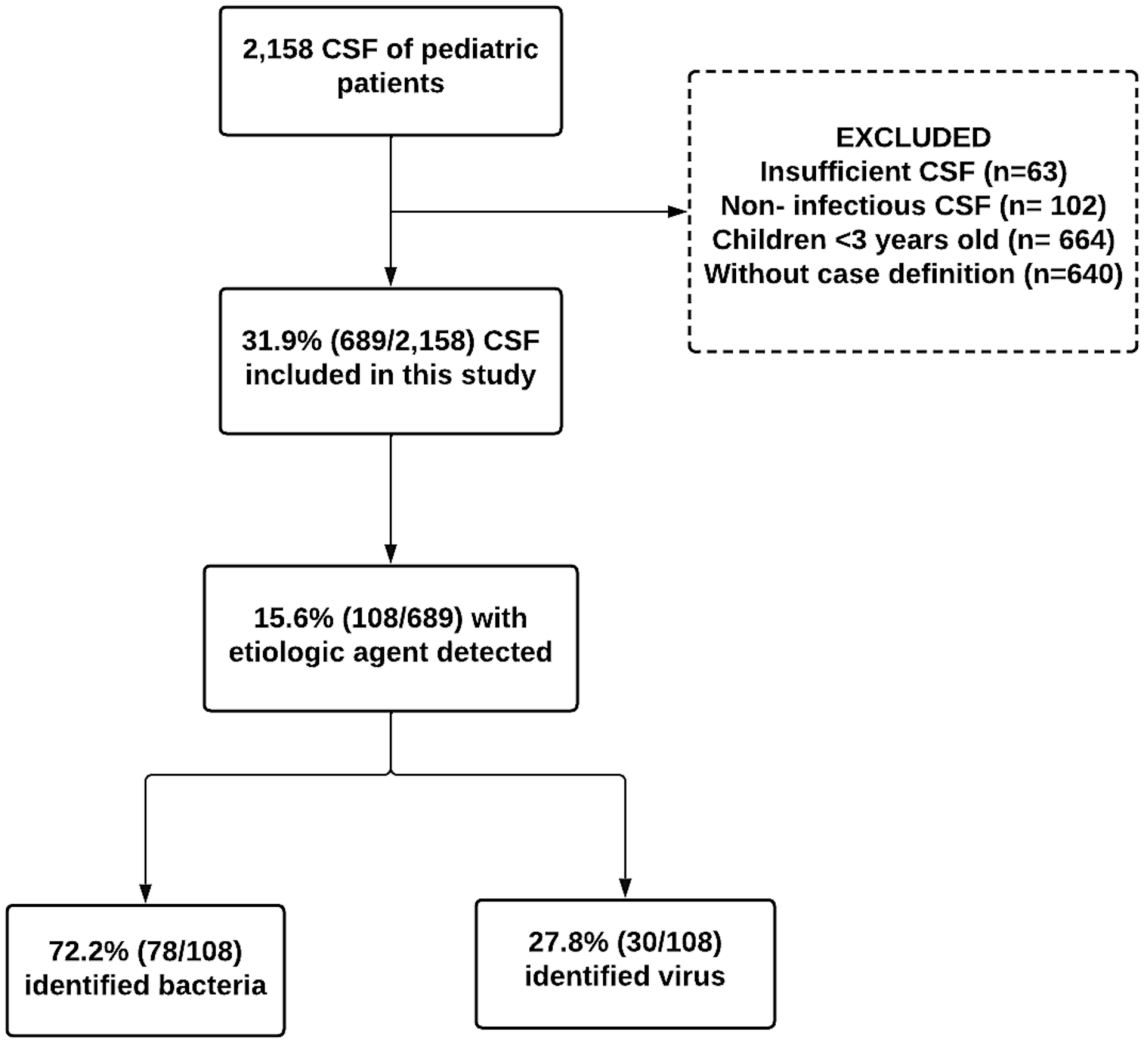
Flowchart of capture, inclusion, and exclusion according to the steps of this 108 research to compose the final sample of pediatric patients with etiological agents detected in the cerebrospinal fluid from Manaus, Amazonas, 2014–2019.

During the study period, 2,158 CSF samples from pediatric patients with suspected CNS infection were evaluated. Out of these, 1,469 samples were excluded for the following reasons: (1) insufficient samples quantity (*n* = 63); (2) cases involving non-infectious CSF, external ventricular shunt, or ventricle-peritoneal shunt, and trauma (*n* = 102); (3) children under 3 months of age without clinical information (*n* = 664); (4) patient not meeting the defined case criteria (*n* = 640). As a result, 689 samples were included in our analysis (Figure 1).

### Demographics

**Figure 2:**
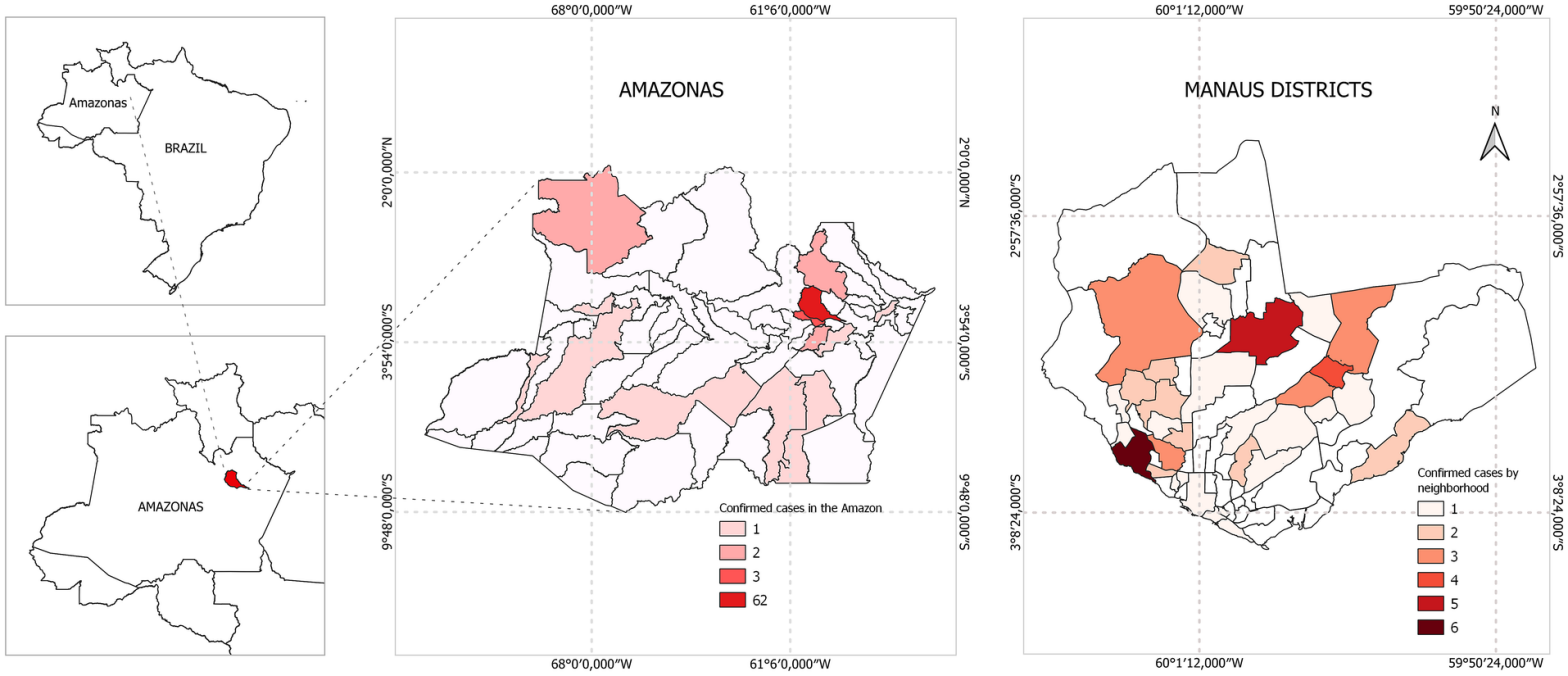
Map of the neighborhoods of the city of Manaus, and the distribution of cases confirmed with etiological agents detected in the cerebrospinal fluid from Manaus, Amazonas, 2014–2019.

Based on our case definition, 108 (15.6%) pediatric patients met our criteria, the mean age was 8 years [SD: 4.8]. Among them, 61.1% of the participants were male, and the most prevalent age group was 10 to 15 years (Table 1). Most of the patients (78.5%) were resident in urban area of Manaus, while 21.5% came from other municipalities of the Amazonas state (S1 Appendix in Supplementary material; Figure 2).

**Table 1:** Baseline characteristics of patients with a central nervous system infection and laboratory-confirmed viral and bacterial infection in cerebrospinal fluid (2014–2019).

Characteristics	Total	Confirmed bacterial infection	Confirmed viral infection
	*n* = 108 (%)	*n* = 78 (%)	*n* = 30 (%)
Male	66 (61.1)	49 (62.8)	19 (63.3)
Female	42 (38.9)	29 (37.2)	13 (33.7)
Age group			
< 1 year	20 (18.0)	15 (19.1)	5 (16.6)
1–4 years	28 (26.0)	20 (25.6)	8 (26.6)
5–9 years	26 (24.0)	18 (23.0)	7 (23.5)
10–15 years	35 (32.0)	25 (32.3)	10 (33.3)

### Clinical and laboratory data

Forty-five percent of the patients required hospitalization, with a median hospital stay of 9 days [range: 1–40 days]. Thirteen children required intensive care, and the average length of stay in the intensive care unit was nine days, which ranged from 1 to 17 days. Furthermore, 39.8% (43/108) of the patients had received prior treatment, with ceftriaxone and penicillin being the most administered antibiotics. Of the patients with a viral infection, nine were treated with both antibiotics and acyclovir. Common symptoms in both bacterial and viral infections included fever, vomiting, headache, and neck stiffness. Notably, altered levels of consciousness were significantly more prevalent (33.3%) in viral infections (Table 2). These data were confirmed by univariate analysis which showed that children with a viral infection (OR: 7.23; CI 95%: 2.23–23.8; *p* = 0.0009) were more likely to have an altered level of consciousness. Death was only recorded in three cases of bacterial infection.

**Table 2:** Signs and symptoms of patients with a central nervous system infection and laboratory-confirmed viral and bacterial infection in cerebrospinal fluid 2014–2019.

Symptoms/Variables	Total	Bacterial	viral	p*
	*n* = 108 (%)	*n* = 78 (%)	*n* = 30 (%)	
Fever	58 (53.7)	39 (50.0)	19 (63.3)	0.300
Vomiting	45 (41.6)	34 (43.5)	11 (36.6)	0.660
Headache	39 (36.1)	25 (32.0)	13 (43.3)	0.380
Neck stiffness	19 (17.5)	14 (17.9)	5 (16.6)	0.900
Altered level of consciousness	15 (13.8)	5 (6.4)	10 (33.3)	0.001
Seizures	9 (8.3)	4 (5.1)	5 (16.6)	0.060

^*^Chi-square test.

### CSF diagnosis

#### Bacterial infections

**Figure 3:**
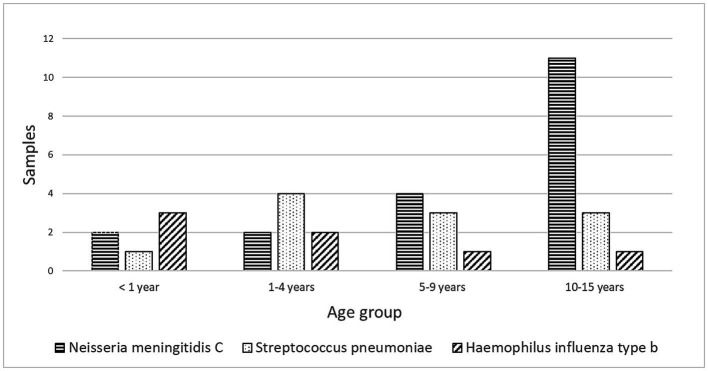
Distribution of Vaccine-preventable bacterial agents isolated in cerebrospinal fluid samples by age group of pediatric patients from Manaus, Amazonas, 2014–2019.

Of the children with confirmed causes of meningitis, 72.2% (78/108) had BM. The CSF culture was positive in 57 patients (73%), and Gram staining identified pathogens in 27% of the CSF samples. Among the bacterial isolates, thirty-seven (64.9%) were vaccine-preventable. *N. meningitidis* serogroup C accounted for 24% of cases, primarily affecting children aged 10 to 15. *S. pneumoniae* constituted 14% of cases, primarily in children aged 1 to 4. *H. influenzae* type b represented 9% of cases, primarily affecting children under 1 year of age. In addition, *Staphylococcus* sp. and Enterobacterales were isolated from 11 and 7 CSF samples, respectively, distributed among different species, including *Serratia* sp. (3 cases), *Pseudomonas* sp. (2 cases), and *Klebsiella pneumoniae*, *Proteus vulgaris*, *P. mirabilis*, and *Acinetobacter* sp. (1 case each) (see Figure 3).

#### Viral infections

Different diagnostic methods identified viral agents in 27.8% (30/108) of the CSF samples, using both molecular and serological techniques (Table 3). Enteroviruses were the primary viral agents detected in 43.3% of the children, occurring in conjunction with meningitis in six patients and encephalitis in five. Only one enterovirus case was initially treated as unspecified BM with prior ceftriaxone treatment. In this patient, CSF protein, glucose, and lactate levels were normal, but cellularity was elevated (300 cells/mm3). Seven children with neurological symptoms were diagnosed with viruses from the Herpesviridae family. Two cases of encephalitis were caused by VZV and EBV, four cases of viral meningitis were caused by VZV, CMV and one case of meningoencephalitis was caused by EBV. In this group, the CSF was clear in four patients and turbid in three, with no evidence of a traumatic puncture. Median CSF parameters were 192 cells/mm3 (range: 0–906 cells/mm3), lymphocytes 98% (range: 5–100), protein 39 mg/dL (range: 0–1,394 mg/dL) and glucose 59 mg/dL (range: 28–83 mg/dL).

**Table 3:** Frequency of viral agents identified in cerebrospinal fluid samples from pediatric patients from Manaus, Brazil (2014–2019).

Viral agents	N°	%
Enterovirus	13	45.0
Varicella zoster virus	2	7.0
Epstein–Barr virus	2	7.0
Cytomegalovirus	1	3.0
Dengue fever	4	13.0
Zika virus	3	10.0
Parvovirus B19	4	16.0
Total	30	100

N simple absolute frequency; %: relative frequency.

PVB-19 was detected in the CSF of 4 (13.3%) patients and was associated with one case of enterovirus. Two patients suffered status epilepticus. Three patients had anemia with hemoglobin levels below 10 g/dL. Five samples tested positive for dengue IgM in ELISA in serum and paired CSF. Two patients were diagnosed with ZIKV via qPCR. Dengue virus was associated with three cases of meningitis and two cases of encephalitis. Notably, one child with ascending acute flaccid paralysis and areflexia required neurological rehabilitation due to severe symptoms.

#### CSF profile

Comparing bacterial and viral infections, we observed significant differences in CSF cellular counts and biochemical profiles (*p* < 0.05). Bacterial infections displayed an inflamed CSF profile with high cellular counts (median 850 cells/mm3, range 10–2,000), predominantly polymorphonuclear, elevated protein levels (median 193 mg/dL), and decreased glucose levels (median 10 mg/dL). In contrast, viral infections showed a moderately altered CSF profile, with a median cellular count of 95.5 cells/mm3, mainly lymphocytes (8–906), a median protein of 62.5 mg/dL, and glucose levels within the reference range (58 mg/dL) (Table 4).

**Table 4:** Cellular and biochemical parameters of cerebrospinal fluid samples from pediatric patients with neurological infection diagnosed at a reference laboratory in Manaus, Brazil, from January 2014 to December 2019.

Biochemical parameters	Bacterial infection (Md)	Viral infection (Md)	*p*
Cytometry (Cell/mm^3^)	850 (10–22.000)	95.5 (0–848)	<0.05
Glucose (mg/dL)	10 (10–91)	58 (17–83)	<0.05
Proteins (mg/dL)	193 (0–5.379)	62.5 (0–3.830)	<0.05
Lactate (mmol/L)	4.5 (0–9.2)	5.0 (0–100)	<0.05

Md = median.

## Discussion

In this hospital-based study, we present data on the etiological agents responsible for CNS infections in pediatric patients hospitalized in Manaus, the capital of the state of Amazonas. Our findings reveal that 15.6% of the pediatric patients were diagnosed with a CNS infection, with *N. meningitidis* serogroup C being the most common bacterial pathogen, followed by *S. pneumoniae*. Among viral pathogens, enterovirus and herpesvirus were prevalent.

We observed that bacterial identification through Gram staining was possible in only 27% of cases, likely due to prior antibiotic use before hospital admission. This underscores the importance of combining molecular diagnostic methods with traditional techniques such as Gram staining and culture to enhance the identification of bacterial pathogens causing CNS infections.

While the use of molecular diagnosis improved the detection of viral pathogens in our study, a low detection rate was noted, consistent with findings in other studies (19). This may be attributed to prior medication usage, which might have eliminated the agent, resulting in undetectable nucleic acid, or due to infections by pathogens not covered by local diagnostic panels.

Our study also supports existing literature indicating that male children are more susceptible to neurological infections, with 61.1% of the children in our study being boys (20).

Clinical signs and symptoms for bacterial and viral infections overlapped, making it crucial to consider both clinical and laboratory findings for accurate diagnosis. Symptoms such as fever, vomiting, and headache were common in both types of infections, while altered consciousness was more pronounced in viral infections. Our findings are in line with previous reports attributing altered consciousness to viral etiologies (21, 22).

Vaccination has altered the epidemiological profile of BM over the past 25 years, reducing the prevalence of vaccine-preventable bacterial infections in pediatric patients. In the 1980s, pathogens like *H. influenzae type* b, *N. meningitidis*, and *S. pneumoniae* were responsible for over 80% of childhood bacterial meningitis cases (23). The incidence of this condition varies by geographic region and is notably higher among pediatric populations (24). Recent data from the National Immunization Program (NIP) in Amazonas reveals vaccination coverage rates for meningococcal C conjugate, pneumococcal, and triple bacterial vaccines for children under 1 year falling below 80%. In our study, we found that 72.2% of CNS infections were bacterial in origin, which aligns with the existing literature’s emphasis on the prevalence of vaccine-preventable bacterial infections in pediatric patients (25). Despite vaccination programs, our findings indicate ongoing circulation of vaccine-preventable bacteria causing CNS infections, highlighting insufficient vaccination coverage in the region, and contributing to the rise in identified cases. These findings underscore the importance of ongoing surveillance for nervous system infections and contribute to future assessments, the development of new diagnostic tests, and our understanding of the impact of vaccination in the pediatric population.

However, other bacteria, such as *Escherichia coli* and *K. pneumoniae*, have emerged as causes of BM, raising concerns about antimicrobial resistance (26, 27). Early antibiotic therapy is vital for treating CNS infections, and broad-spectrum cephalosporins are recommended until the causative agent and its susceptibility are determined (28). In our study, ceftriaxone was the most used antibiotic.

Molecular methods have advanced the diagnosis of neurological infections, increasing the detection of viral pathogens in CSF. Among the main etiological agents related to CNS infections are the enteroviruses, followed by viruses of the family Herpesviridae and the dengue virus (29). Enteroviruses are responsible for the majority cases of CNS infections, especially among the pediatric population (30). Although EV infections are usually asymptomatic, they can also be associated with a variety of clinical manifestations and severe CNS syndromes, such as encephalitis, meningitis, and acute flaccid paralysis (31). Our data indicates that Enteroviruses were the most prevalent viral pathogens, often associated with meningitis and encephalitis, which is consistent with previous studies (32, 33).

Antimicrobial treatments in cases of CNS infections are, in general, crucial for improving clinical outcomes, but their empirical application plays a crucial role in the evolution of resistance genes (34). In our study, we found one case of enterovirus meningitis, which was treated with antibiotic. Therefore, we emphasize the importance of a differential, rapid and assertive diagnosis in order to reduce the exposure of patients to unnecessary antimicrobial therapy.

PVB-19 has been linked to various syndromes, including erythematous or asymptomatic febrile infections (35, 36). However, the neurological manifestations associated with PVB-19 have not been extensively investigated. In our study, we detected PVB-19 in the CSF of four children without skin manifestations. These children did not exhibit any signs of rash, but three of them had anemia and aplastic anemia. It is noteworthy that neurological PVB-19 infections without skin involvement have been well-documented in the literature (37, 38). Furthermore, it has also been suggested that this virus may be associated with pancytopenia and erythrocyte plasticity (39). With the growing number of reports highlighting neurological manifestations in regions where PVB19 is prevalent (40), it is crucial to emphasize the importance of considering this virus in investigations of neurological diseases.

Herpesviridae is a substantial family of DNA viruses that can potentially induce neurological diseases either during the primary infection or upon reactivation in immunosuppressive conditions (41). In our study, we identified herpesviruses, including VZV, CMV, and EBV, in 17% of CSF samples. These viruses were associated with a range of neurological manifestations such as meningitis, encephalitis, and meningoencephalitis. These findings align with those of Imamba and colleagues (42), who observed a similar pattern of herpesvirus-related meningitis and encephalitis as predominant conditions in children admitted to a hospital in Zambia.

Arboviruses, such as including dengue, Zika and chikungunya, are commonly found in Brazil, and are emerging pathogens known to cause both febrile and neurological syndromes in the general population (43). Consequently, the differential diagnosis of neurological syndromes has become increasingly vital in regions where these viruses are endemic (44). While Flaviviruses are typically associated with acute febrile infections characterized by skin rashes, they can also trigger neuroinflammation, leading to neurological disorders like meningitis, encephalitis, and meningoencephalitis (45). In our study, we identified Flaviviruses in seven cases (23%) of the suspected CNS viral infection. Notably, DENV was associated with cases of meningitis and encephalitis in pediatric patients. Our findings are consistent with those of Marinho et al. (46), who also reported a low prevalence of dengue in CNS samples from the pediatric population. In arbovirus-endemic regions like the Amazon, it is important laboratory surveillance for monitoring the diagnosis of encephalitogenic viruses.

The altered CSF profile provides evidence of a bacterial infection through the presence of a pleocytosis characterized by a predominance of neutrophils, low glucose levels, and high-protein levels (47). Viral infections, on the other hand, exhibit distinct presentations: mononuclear pleocytosis, moderate protein elevations, and normal glucose levels (48). Notably, our study reveals statistically significant differences in cellular and biochemical results within the CSF between bacterial and viral infections, indicating variations in cellularity, protein, and glucose levels. These findings align with the research conducted by Nazir et al. (49), emphasizing the greater alterations in CSF profiles for bacterial infections and underscoring the importance of measuring lactate levels in CSF as a crucial marker for distinguishing between bacterial and viral infections.

CSF examinations, being cost-effective and readily accessible, play a pivotal role in assessing both bacterial and viral infections. Particularly in resource-limited regions like the Amazon, where laboratory infrastructure is limited, reliance on field-testing is common. Establishing a marker such as lactate has the potential to significantly enhance clinical management.

This study has limitations due to a lack of information on risk factors and selection bias. Data analysis based on disease severity and outcome for recovery or death was not possible due to this being a retrospective analysis of laboratory data and medical records. Despite the small number of samples between bacterial and viral infection groups, we cannot rule out the lack of power in comparisons between signs and symptoms. We were unable to obtain information on antibiotic therapy for all the patients and their treatment period. In addition, it was impossible to retrieve information about the vaccination status of children. As the bacteriology laboratory of FMT-HVD analyzed samples that were sent by other services, the total number of infections in the CNS of patients in the Manaus city may have been underestimated.

In conclusion, our study provides valuable insights into the frequency and etiology of CNS infections in pediatric patients in Manaus. This information can improve future diagnostic approaches, the impact of vaccination, and public health planning to prevent nervous system infections in this population. Despite vaccination efforts, vaccine-preventable bacteria continue to circulate, emphasizing the need for ongoing monitoring and prevention strategies.

## Data availability statement

The raw data supporting the conclusions of this article will be made available by the authors, without undue reservation.

## Ethics statement

The studies involving humans were approved by the Ethics Review Board of the Fundação de Medicina Tropical Heitor Vieira Dourado. The studies were conducted in accordance with the local legislation and institutional requirements. The human samples used in this study were acquired from primarily isolated as part of your previous study for which Ethical Approval was obtained. Written informed consent for participation was not required from the participants or the participants’ legal guardians/next of kin in accordance with the national legislation and institutional requirements.

## Author contributions

EM: Conceptualization, Investigation, Writing – original draft, Writing – review & editing. EF: Formal analysis, Writing – review & editing. CB: Investigation, Writing – review & editing. SM: Investigation, Methodology, Writing – review & editing. JC: Data curation, Formal analysis, Writing – review & editing. SP: Investigation, Writing – review & editing. RM: Investigation, Supervision, Visualization, Writing – review & editing. VSi: Investigation, Methodology, Writing – review & editing. YM: Investigation, Writing – review & editing. MR: Supervision, Validation, Writing – review & editing. SS-G: Supervision, Writing – review & editing. VSa: Supervision, Writing – review & editing, Data curation, Formal analysis. DC: Writing – review & editing, Supervision, Validation. PF: Methodology, Writing – review & editing, Investigation. LR: Supervision, Writing – review & editing, Methodology. LF: Funding acquisition, Supervision, Writing – review & editing, Validation. MB: Conceptualization, Funding acquisition, Supervision, Writing – original draft, Writing – review & editing.
