# Supplementary material for: Pediatric central nervous system infections in the Amazon: clinical and laboratory profiles
Source: Front Public Health. 2023 Dec 21;11:1329091. doi: 10.3389/fpubh.2023.1329091 (PMC10768178; doi:10.3389/fpubh.2023.1329091)
Supplement: Supplementary file 1 [file Table_1.DOCX]

**S1 Appendix**

**Hospitals:**

Fundação Centro de Controle de Oncologia do Estado do Amazonas – FCECON

Fundação de Medicina Tropical Dr. Heitor Vieira Dourado – FMT-HVD

Hospital e Pronto Socorro Dr Aristóteles Platão Bezerra de Araújo

Hospital Santa Júlia

Hospital Infantil Dr. Fajardo

Hospital e Pronto Socorro da Criança – Joãozinho

Hospital e Pronto-Socorro da Criança Zona Sul

Hospital e Pronto-Socorro da Criança Zona Oeste

Hospital e Pronto-Socorro Delphina Rinaldi Abdel Aziz

Maternidade Ana Braga

UNIMED

**Municipalities**

Autazes

Benjamin Constant

Careiro

Iranduba

Jutaí

Novo Aripuanã

Presidente Figueiredo

São Gabriel da Cachoeira

Tapauá

Tonantins

**Table 4**: Cellular and biochemical parameters of cerebrospinal fluid samples from pediatric with neurological infection diagnosed at a reference laboratory in Manaus, Brazil, from January 2014 to December 2019.

Kruskal-Wallis Test

Cytometry

| **H =** | 25.1761 |  |  |  |
| --- | --- | --- | --- | --- |
| **Degrees of freedom =** | 1 |  |  |  |
| **(p) Kruskal-Wallis =** | < 0.0001 |  |  |  |
| **R 1 =** | 4982.5000 |  |  |  |
| **R 2 =** | 903.5000 |  |  |  |
| **R 1 =** | 63.8782 |  |  |  |
| **R 2 =** | 30.1167 |  |  |  |
| **Comparisons (Dunn’ method)** | **Dif. Positions** | **z calculated** | **z critical** | ***p*** |
| **Medium Posts** **1 e 2** | 33.7615 | 5.0175 | 1.96 | < 0.05 |

Protein

| **H =** | 28.1860 |  |  |  |
| --- | --- | --- | --- | --- |
| **Degrees of freedom =** | 1 |  |  |  |
| **(p) Kruskal-Wallis =** | < 0.0001 |  |  |  |
| **R 1 =** | 5025.0000 |  |  |  |
| **R 2 =** | 861.0000 |  |  |  |
| **R 1 =** | 64.4231 |  |  |  |
| **R 2 =** | 28.7000 |  |  |  |
| **Comparisons (Dunn’ method)** | **Dif. Positions** | **z calculated** | **z critical** | ***p*** |
| **Medium Posts** **1 e 2** | 35.7231 | 5.3090 | 1.96 | < 0.05 |

Glucose

| **H =** | 30.0085 |  |  |  |
| --- | --- | --- | --- | --- |
| **Degrees of freedom =** | 1 |  |  |  |
| **(p) Kruskal-Wallis =** | < 0.0001 |  |  |  |
| **R 1 =** | 3453.0000 |  |  |  |
| **R 2 =** | 2433.0000 |  |  |  |
| **R 1 =** | 44.2692 |  |  |  |
| **R 2 =** | 81.1000 |  |  |  |
| **Comparisons (Dunn’ method)** | **Dif. Positions** | **z calculated** | **z critical** | **p** |
| **Medium Posts** **1 e 2** | 36.8308 | 5.4736 | 1.96 | < 0.05 |

Lactate

| **H =** | 40.8366 |  |  |  |
| --- | --- | --- | --- | --- |
| **Degrees of freedom =** | 1 |  |  |  |
| **(p) Kruskal-Wallis =** | < 0.0001 |  |  |  |
| **R 1 =** | 5182.5000 |  |  |  |
| **R 2 =** | 703.5000 |  |  |  |
| **R 1 =** | 66.4423 |  |  |  |
| **R 2 =** | 23.4500 |  |  |  |
| **Comparisons (Dunn’ method)** | **Dif. Positions** | **z calculated** | **z critical** | **p** |
| **Medium Posts** **1 e 2** | 42.9923 | 6.3893 | 1.96 | < 0.05 |
